# Complementary Network-Based Approaches for Exploring Genetic Structure and Functional Connectivity in Two Vulnerable, Endemic Ground Squirrels

**DOI:** 10.3389/fgene.2017.00081

**Published:** 2017-06-14

**Authors:** Victoria H. Zero, Adi Barocas, Denim M. Jochimsen, Agnès Pelletier, Xavier Giroux-Bougard, Daryl R. Trumbo, Jessica A. Castillo, Diane Evans Mack, Mark A. Linnell, Rachel M. Pigg, Jessica Hoisington-Lopez, Stephen F. Spear, Melanie A. Murphy, Lisette P. Waits

**Affiliations:** ^1^Haub School of Environment and Natural Resources, University of WyomingLaramie, WY, United States; ^2^Department of Zoology and Physiology, University of WyomingLaramie, WY, United States; ^3^Program in Ecology, University of WyomingLaramie, WY, United States; ^4^Department of Biological Sciences, University of IdahoMoscow, ID, United States; ^5^Department of Environmental Studies and Sciences, University of WinnipegWinnipeg, MB, Canada; ^6^Department of Natural Resource Sciences, McGill UniversityMontreal, QC, Canada; ^7^School of Biological Sciences, Washington State UniversityPullman, WA, United States; ^8^Department of Fisheries and Wildlife, Oregon State UniversityCorvallis, OR, United States; ^9^Idaho Department of Fish and Game, McCall SubregionMcCall, ID, United States; ^10^Division of Biology, Kansas State UniversityManhattan, KS, United States; ^11^The Edison Family Center for Genome Sciences and Systems Biology, Washington University School of MedicineSt. Louis, MO, United States; ^12^The WildsCumberland, OH, United States; ^13^Department of Ecosystem Science and Management, University of WyomingLaramie, WY, United States; ^14^Department of Fish and Wildlife Sciences, University of IdahoMoscow, ID, United States

**Keywords:** functional connectivity, gene flow, graph theory, gravity model, landscape genetics, Sciuridae, *Urocitellus* [Spermophilus]

## Abstract

The persistence of small populations is influenced by genetic structure and functional connectivity. We used two network-based approaches to understand the persistence of the northern Idaho ground squirrel (*Urocitellus brunneus)* and the southern Idaho ground squirrel (*U. endemicus*), two congeners of conservation concern. These graph theoretic approaches are conventionally applied to social or transportation networks, but here are used to study population persistence and connectivity. Population graph analyses revealed that local extinction rapidly reduced connectivity for the southern species, while connectivity for the northern species could be maintained following local extinction. Results from gravity models complemented those of population graph analyses, and indicated that potential vegetation productivity and topography drove connectivity in the northern species. For the southern species, development (roads) and small-scale topography reduced connectivity, while greater potential vegetation productivity increased connectivity. Taken together, the results of the two network-based methods (population graph analyses and gravity models) suggest the need for increased conservation action for the southern species, and that management efforts have been effective at maintaining habitat quality throughout the current range of the northern species. To prevent further declines, we encourage the continuation of management efforts for the northern species, whereas conservation of the southern species requires active management and additional measures to curtail habitat fragmentation. Our combination of population graph analyses and gravity models can inform conservation strategies of other species exhibiting patchy distributions.

## Introduction

Habitat loss and fragmentation are threats to many species of conservation concern (Wilcox et al., 1985; Groombridge, 1992). These agents of landscape change decrease the size and structural connectivity of habitat patches, with consequences for long-term population viability and species distributions (Kareiva and Wennergren, 1995; Fahrig, 2002). Decreased animal movement, and subsequent reduction in gene flow, can lead to isolated populations and constricted species ranges (Andrews, 1990; Yahner and Mahan, 1997; Fahrig, 2002). Over time, reduced gene flow can decrease population size, alter population dynamics, and lower persistence probability (Meffe and Carroll, 1997; Ovaskainen and Hanski, 2003). Isolated populations typically have low levels of genetic variation (Frankham, 1997) that inhibit adaptation in the face of environmental change (Lande, 1988) and increase vulnerability to inbreeding depression (Frankham, 1995; Hedrick, 2005) and local extinction (Burkey, 1995; Frankham et al., 2002).

As the long-term persistence of populations in fragmented landscapes depends on functional connectivity, or how individuals respond to landscape composition (Tischendorf and Fahrig, 2000; Stevens et al., 2006), research that assesses the effects of landscape and ecological features on gene flow serves as a valuable conservation tool (McRae et al., 2008). The spatial context and composition of habitat patches generally have profound influences on animal movement beyond the effect of geographical distance alone (Ricketts, 2001). Landscape genetic methods are particularly suited to test how environmental context influences patterns of genetic variation and gene flow across temporal and spatial scales (Manel et al., 2003; Storfer et al., 2007; Holderegger and Wagner, 2008), and have recently been strengthened by the integration of graph theoretic approaches (Garroway et al., 2008; Murphy et al., 2016). These approaches provide a mathematical framework in which researchers can represent populations or sites as “nodes” and connections between them as “edges,” and then evaluate patterns of connectivity to identify environmental factors underlying gene flow (Dyer and Nason, 2004; Garroway et al., 2008; McRae et al., 2008; Dyer et al., 2010; Murphy et al., 2010).

Graph theory can be used to assess functional connectivity, and may therefore provide important information for conservation planning. Network metrics such as degree centrality and betweenness (Everett and Borgatti, 2005) measure the relative contribution of sampled sites to overall population connectivity, and thus can pinpoint the best locations for conservation or management actions. Gravity models (Fotheringham and O'Kelly, 1989) can simultaneously evaluate the relative influence of geographic distance, local attributes of sampling locations (at-site characteristics), and the features that separate them (between-site characteristics) on gene flow (Murphy et al., 2010). Typical landscape genetic network models do not include the influence of local attributes. By including at-site characteristics in these models, we can incorporate additional factors contributing to gene flow by quantifying how habitat patches differ in quality (Ovaskainen and Hanski, 2003). Patches of higher quality habitat may produce more offspring and thereby contribute disproportionately to gene flow. Gravity models can help determine how landscapes should be managed to maintain connectivity and improve patch quality, and network metrics can identify where managers should focus conservation efforts.

The northern Idaho ground squirrel (*Urocetillus brunneus*; NIDGS) and the southern Idaho ground squirrel (*U*. *endemicus*; SIDGS) are two congeners of conservation concern. NIDGS and SIDGS are endemic to west-central Idaho and were originally classified as two subspecies (Yensen, 1991) but were recently elevated to distinct species based on genetic differences (U.S. Fish and Wildlife Service, 2015), morphology, behavior, and distinct geographic and ecological niches (Hoisington-Lopez et al., 2012). These species occur in small, discrete populations within a fragmented landscape (Van Horne et al., 2007; Yensen et al., 2008). Consequently, population graph analysis and gravity models can lend insight into factors affecting their population connectivity.

Their ranges are restricted and fragmented; both species have experienced population declines and reductions in the number and total area of sites occupied (Sherman and Runge, 2002; U.S. Fish and Wildlife Service, 2003; Yensen et al., 2008; Lohr et al., 2013). In recent years, the number of occupied locations and subpopulations has remained relatively stable, while the number of mature individuals appears to fluctuate according to several-year cycles (Evans Mack, personal communication). For example, between 2011 and 2016, overall population size ranged between just under 1,000 and over 2,500 individuals. Consequently, the United States Fish and Wildlife Service listed NIDGS as threatened in 2000 (Clark, 2000), while SIDGS was a candidate for listing until just recently (Federal Register, November 22, 2013 Vol. 68, No. 226:77 70103-7016). Primary threats to NIDGS include the loss of preferred habitat to ponderosa pine (*Pinus ponderosa*) encroachment due to fire suppression (Yensen and Sherman, 1997; Gavin et al., 1999; Sherman and Runge, 2002), and competition with the Columbian ground squirrel (*Urocitellus columbianus*; Dyni and Yensen, 1996). The latter species occurs throughout central Idaho, potentially overlapping populations of both Idaho ground squirrels. Declines in SIDGS are attributed to the invasion of non-native annual plants, including cheatgrass (*Bromus tectorum*) and medusahead (*Taeniatherum asperum*), which have increased fire frequency and intensity with subsequent shifts in vegetation composition (Yensen, 1991; Lohr et al., 2013).

The loss and degradation of preferred habitat have consequences for the long-term persistence of remaining NIDGS and SIDGS populations. Population divergence has been detected for NIDGS using allozymes (Gavin et al., 1999), and for both species using mitochondrial DNA (Yensen and Sherman, 1997; Garner et al., 2005; Hoisington-Lopez et al., 2012) and microsatellite data (NIDGS: 0.03 < F_ST_ < 0.46; SIDGS: 0.04 < F_ST_ < 0.43; Garner et al., 2005; Hoisington-Lopez et al., 2012). In addition, both species have low to moderate levels of genetic diversity (allelic richness, expected heterozygosity, and haplotype diversity; Garner et al., 2005; Hoisington-Lopez et al., 2012) that are likely a consequence of isolation and bottleneck events. The effects of landscape and environmental variables on genetic diversity and connectivity of NIDGS and SIDGS have not been evaluated in depth. Understanding the ecological drivers underlying site productivity and factors facilitating gene flow among habitat patches is a critical conservation need for both species. Identifying sites that contribute the most to functional connectivity is also essential for making conservation and management decisions.

Our primary goal was to quantify functional connectivity among NIDGS and SIDGS populations and identify sites contributing the most to gene flow to help inform conservation and management efforts. We aimed to evaluate functional connectivity for each species using genetic patterns and identify at-site and between-site variables influencing gene flow. We hypothesized that the production of potential migrants from a site would be affected by forage availability as indicated by local climate measures. For NIDGS, availability of meadow (i.e., grassland) should be an important factor in population connectivity since this preferred habitat has been reduced by forest encroachment. For SIDGS, highly developed areas (measured by impervious surfaces) should reduce functional connectivity due to potential movement barriers and the likely increased incidence of non-native plants species. We also examined topographic complexity, waterways, soils, and competition from Columbian ground squirrels (*U. columbianus*) as potential drivers of functional connectivity.

## Materials and methods

### Study area and species

**Figure 1:**
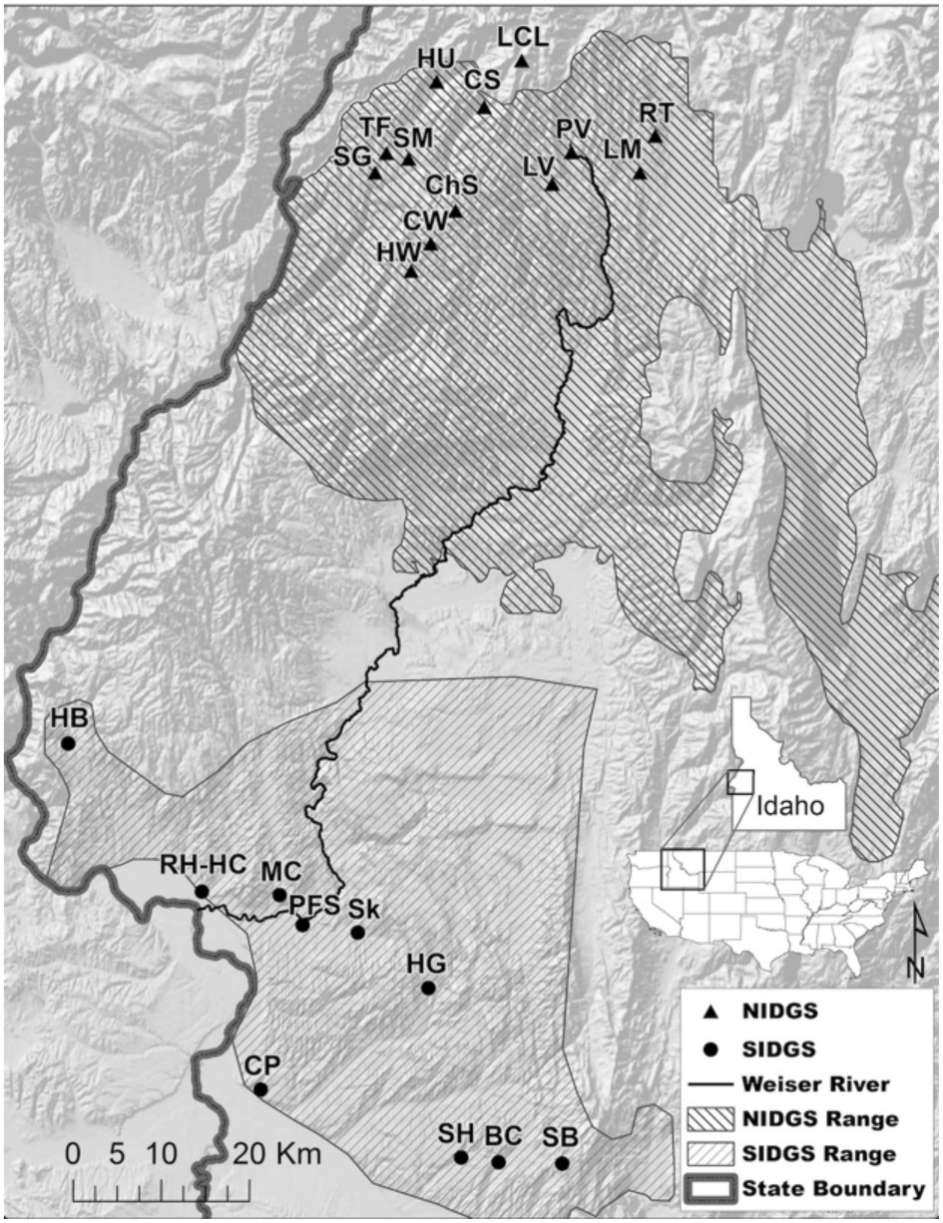
Sampling locations of genetic data for northern Idaho ground squirrel (*Urocetillus brunneus*; NIDGS) and southern Idaho ground squirrel (*U.endemicus*; SIDGS). Also shown are the NIDGS probable historic distribution (U.S. Fish and Wildlife Service, 2003) and the current known range for SIDGS (Idaho Game and Fish Department). Individuals were sampled from 2002 to 2006 (Hoisington-Lopez et al., 2012). Background hillshade map was produced from the National Elevation Dataset (http://ned.usgs.gov). Full site names and sample sizes can be found in Table S1.

We examined the functional connectivity of northern and southern Idaho ground squirrel populations from 23 sites within 5 counties located in west-central Idaho (Figure 1, Table S1). No new field or genetic data were collected for this study. All procedures for initial data collection were approved by the University of Idaho Animal Care and Use Committee (2006-35), Idaho Fish and Game state permit (060308), and federal permit for *U. brunneus* (subpermit FWSSRBO-5). Extant, sampled NIDGS and SIDGS populations were previously determined by methods described by Yensen (1991). Mean sampling location area (±SE) was 0.44 ± 0.21 km^2^ for NIDGS and 0.42 ± 0.11 km^2^ for SIDGS. The study area includes the geographically discrete ranges of both species, extending between the Salmon and Payette Rivers. NIDGS inhabit mid to high elevations (1,150–2,300 m) in xeric, montane meadows, and grasslands surrounded by coniferous forests (Yensen, 1991; Yensen and Sherman, 1997). SIDGS occur at lower elevations (670–975 m) in sagebrush and bitterbrush habitats with interspersed perennial bunchgrasses and forbs (Hafner et al., 1998; IDFG unpublished data). The majority of habitat is under public ownership, with private land primarily at lower elevations (U.S. Fish and Wildlife Service, 2013). Land use includes logging, agriculture, grazing, and suburban developments (Yensen et al., 2008).

### Genetic data

We obtained multilocus, microsatellite genotypes from previous studies that, cumulatively, sampled most IDGS populations comprised of more than 10 individuals (Figure 1; Supplementary Data Sheet 1, Garner et al., 2005; Hoisington-Lopez et al., 2012). NIDGS sampling occurred in 2002 and 2006, and included 316 individuals from 13 locales (Table S1; Hoisington-Lopez et al., 2012). We excluded one NIDGS population from this study (Round Valley), as previous results indicate that it is both geographically and genetically isolated from all other NIDGS populations, and thus could lead to spurious correlations with landscape variables (Cushman and Landguth, 2010). SIDGS sampling consisted of 263 individuals in 2002 and 2006 from 11 locations (Table S1). When samples were collected at the location in multiple years, we tested for differences in allele frequency distributions before combining data (Hoisington, 2007). We used data from previously published microsatellite loci (*n* = 8) in Hardy–Weinberg equilibrium that showed no linkage disequilibrium (Hoisington-Lopez et al., 2012).

For each species, we calculated three measures of genetic distance between populations to serve as response variables in network models: (1) the proportion of shared alleles (D_PS_; Bowcock et al., 1994), calculated using Microsatellite Analyzer 4.05 (Dieringer and Schlötterer, 2003); (2) conditional genetic distance (cGD; Dyer et al., 2010), using Genetic Studio (Dyer, 2009) in R (gstudio 0.8, R Core Development Team, 2012); and (3) the fixation index (F_ST_), a commonly used measure of population structure, calculated using Fstat (Goudet, 1995). D_PS_ is not subject to the equilibrium assumptions inherent in divergence (F_ST_) measures, and thus may be more appropriate for measuring genetic connectivity among populations subject to recent disturbance, and cGD has been shown to outperform F_ST_ in some situations. Furthermore, cGD focuses only on population pairs that exhibit conditional dependence with one another and thus are likely to be directly exchanging migrants, and ignores population pairs that are conditionally independent and likely not directly exchanging migrants (Dyer et al., 2010). For each species, we additionally performed a Mantel test (Smouse et al., 1986) to examine the correlation of geographic distance with the two relevant metrics of genetic distance, D_PS_ and F_ST_ (Table S2).

### Population graph analysis

To conduct the population graph analyses, we used cGD (Dyer et al., 2010), a metric that calculates the distance between each pair of nodes, thereby accounting for the genetic covariance in the whole network. The method examines pairwise correlations in inverted cGD values among sampling locations and draws an edge between two nodes if the partial correlation between them is significantly higher than expected by chance. The subsequent pruned graph contains the minimal number of edges which will sufficiently describe the total covariance structure among populations (Dyer et al., 2010). Because pruned networks are more information than saturated networks (Dyer and Nason, 2004), we kept them for subsequent analyses.

To help guide conservation actions, we determined the number of significant genetic units (genetic clusters) using two community detection methods, which identify “communities” of more highly connected nodes (Girvan and Newman, 2002). The first, Girvan-Newman uses an optimization procedure based on eigenvalues to calculate the support for different cluster numbers in terms of modularity (Q; the existence of non-overlapping groups of nodes in the network). The best-supported model of community division receives the highest modularity value (Newman, 2006). The second, the *Walktrap* algorithm, finds subgraphs of more densely connected nodes based on random walks and also calculates overall modularity (Pons and Latapy, 2006). To perform these analyses, we built a binary network for each species.

To determine the relative contribution of each sampling location to overall gene flow, we investigated the network topologies of both species by calculating four network metrics for each node: (1) degree centrality—the number of connections that each node has in the network (Everett and Borgatti, 2005), (2) strength centrality—the sum of all association indices (i.e., weighted connections among nodes) that each node has in the network (Garroway et al., 2008), (3) betweenness—the number of shortest paths that a particular node or edge lies on, which can identify bottlenecks (Everett and Borgatti, 2005), and (4) coreness—an algorithm that tests for the existence of a core/periphery structure in the network and calculates the location of each node in relation to the core. Based on the number of core nodes, we additionally calculated a concentration score (ranging from 0 to 1) which quantifies how close the network is to an idealized core-periphery model, in which all nodes in the core are connected within the core and to the periphery nodes and all nodes in the periphery are not connected (Borgatti and Everett, 1999). In the context of genetic networks, the coreness of a node can be interpreted as the extent to which it acts as a source for dispersing individuals. Sampling location abbreviations are presented in Table S1.

**Figure 2:**
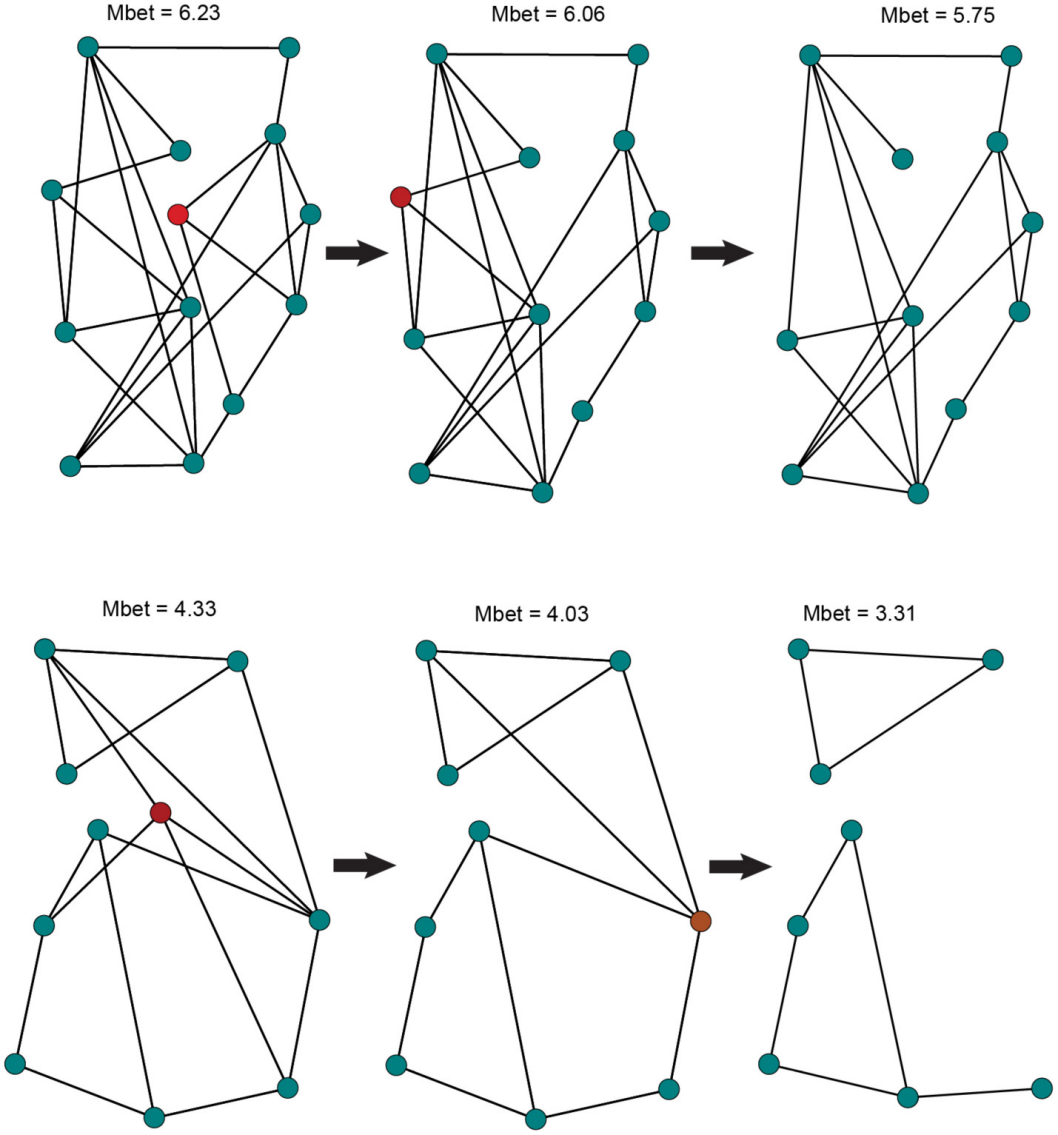
Illustration of the node removal procedure used to simulate population extinction events in NIDGS (top) and SIDGS (bottom). In each step, a randomly selected node (in red), representing a sampling location, is removed from the network along with the edges connecting it to additional nodes. Network mean betweenness values are given on top. Following the removal of 2 nodes, the SIDGS network fails to create a single component, becoming fragmented.

To examine the vulnerability of each species to local extinction, we assessed network sensitivity to node removal (Figure 2). Node removal simulates local patch extinction, a recurrent event in species that exhibit metapopulation structure (Hanski, 1998). We sequentially removed random nodes to generate up to 100 population graphs for each scenario (e.g., 1, 2, 3 nodes removed). For each of the simulated graphs, we assessed overall gene flow using two metrics: (1) Proportion of fully connected graphs, quantifying the extent to which the population graph will become fragmented as a result of node removal; (2) Size of the largest graph component, measuring the maximal number of nodes that retained connectivity among them. We calculated this metric proportional to the total network size. We built 95% confidence intervals, based on standard errors, around the proportional size of the largest component for each node removal scenario.

### Gravity models

We used gravity models (Fotheringham and O'Kelly, 1989; Murphy et al., 2010) to analyze the effects of abiotic and biotic variables on population connectivity. We modeled gene flow [1-genetic distance (D_PS_)] as a function of geographic (Euclidean) distance (*w*), attributes of nodes (*v*), and landscape resistance factors (*c*) that limit or facilitate movement of individuals between nodes (Murphy et al., 2010). We developed a set of *a priori* hypotheses to describe ecologically relevant processes affecting at-site production of migrants and between-site landscape resistance for both species (Table 1).

**Table 1:** Independent variables tested in candidate gravity models to explain functional connectivity of two species of Idaho ground squirrels.

**Parameter**	**Process**	**Variable**	**Code**	**Predicted effect**	**Description**	**Ecological justification**	**Source**	**Species modeled**
*Geographic distance (w)*	IBD	Geographic distance	*w*	−	Euclidean distance (m)	IBD previously detected in SIDGS (Hoisington, 2007).	SRTM	NIDGS^*^
								SIDGS^*^
*At-site production (v)*	Habitat	Vegetation	*shrub, grass*	+	Percent cover meadow (NIDGS) or shrubland (SIDGS)	An increased proportion of meadow habitat could yield larger populations (Hoisington-Lopez et al., 2012).	NLCD	NIDGSSIDGS
		Soil texture	*soil*	+	Percent loam	IDGS are associated with well-drained, loamy soils (Yensen and Sherman, 1997).	NRCS	SIDGS
	Productivity	Heat load index	*hli*	+	Measure of solar intercept as derived from slope aspect (McCune and Keon, 2002)	Slope and aspect influence forage production; data suggest inherent preferences (U.S. Fish and Wildlife Service, 2003; Lohr et al., 2013).	SRTM	NIDGS^*^SIDGS^*^
		Frost-free period	*ffp*	+	Length of frost-free period	Longer frost-free periods indicative of higher plant productivity (Hoisington-Lopez et al., 2012).	Spline	NIDGSSIDGS^*^
		Growing season precipitation	*gsp*	+	Precipitation received from April to September	Annual precipitation important in ecological niche models (Hoisington-Lopez et al., 2012). Growing season precipitation may be more directly related to plant productivity.	Spline	NIDGSSIDGS^*^
	Competition	Columbian ground squirrel distribution	*cogs*	−	Percent overlap of CGS core areas with NIDGS core areas	NIDGS may be competitively inferior in meadows where CGS occur (Yensen, 1991).	IDFG	NIDGS
*Between-site resistance (c)*	Topography	Topographic complexity	*srr3, srr27*	−	Elevation relief ratio (Evans, 1972)	Fine scale topographic complexity (3 × 3) may make movement energetically costly. Large-scale complexity (27 × 27) may represent barriers.	SRTM	NIDGS^*^SIDGS^*^
	Habitat	Land cover shrub, grass	*shrub, grass*	+	Proportion of intervening shrub and grassland	Meadows provide suitable habitat for NIDGS (Yensen and Sherman, 1997). Suitable burrowing and foraging habitat between populations could enhance dispersal opportunities.	NLCD	NIDGSSIDGS
		Land cover agriculture	*agri*	−	Proportion of intervening agricultural and developed areas	Cultivated areas could limit dispersal and are associated with human activity.	NLCD	SIDGS
		Ephemeral streams	*eph_strm*	+	Stream density	Ephemeral and intermittent streams may act as dispersal corridors when dry (Roach et al., 2001).	NHD	NIDGSSIDGS
	Barriers	Impervious surfaces	*imperv*	−	Percent imperviousness	Road traffic may cause mortalities on all road types. Paved roads may represent habitat loss and adjacent areas may be altered.	NLCD	SIDGS^*^
		Perennial streams, rivers	*per_strm*	−	Stream density	Streams and rivers may represent absolute barriers to dispersal. The Weiser River was identified as a barrier to SIDGS gene flow (unpublished data).	NHD	SIDGS

*Parameter: the parameter estimated in the gravity equation [distance (w), production (v), and resistance (c)]. Process: the landscape process influencing gene flow: isolation-by-distance (IBD), habitat quality/permeability, site productivity, interspecific competition, topography, or geographic barriers. Predicted relationship: expectation of a positive (+) or negative (−) relationship between the independent variable and gene flow. Source: source of data containing the variable or used to derive the variable. NLCD, National Land Cover Database (2001); NRCS, Natural Resources Conservation Service; IDFG, Idaho Department of Fish and Game; SRTM, Shuttle Topographic Radar Mission digital elevation model; a climate spline model (Spline; Rehfeldt, 2006), and NHD: National Hydrography Dataset. Species modeled: NIDGS, northern Idaho ground squirrel; SIDGS, southern Idaho ground squirrel. Asterisks indicate variables that were found to be important in gravity models (Table 2)*.

We used 30 m landcover data from the LANDFIRE Existing Vegetation Type dataset, and used our between-site calculations to assess habitat permeability (http://landfire.cr.usgs.gov/viewer). We extracted the landcover data for grassland, shrubland, agriculture, and impervious surfaces (i.e., roads and developed areas). We then calculated percent cover for each cover type within a 90 × 90 pixel moving window. We calculated surface relief ratio (*srr*; Evans, 1972) from 10 m Shuttle Radar Topography Mission digital elevation models using two neighborhood sizes (3 × 3 and 27 × 27 pixels), to assess topographic resistance to gene flow. We used the Geomorphometry and Gradient Metrics Toolbox (http://evansmurphy.wix.com/evansspatial#!arcgis-gradient-metrics-toolbox/crro) in ArcMap 10.2. We tested 6 biotic and abiotic variables hypothesized to affect at-site production/attraction (*v*) of IDGS migrants, such as climate, soil type, vegetation cover, and inter-specific competition. For landscape resistance between sites (*c*), we developed a set of 6 abiotic and biotic variables that relate to habitat permeability, topography, hydrologic complexity, and road density. For between-site variables, we calculated the average or variance along each edge (30 m width) connecting populations in the network. We also tested for the effect of spatial scale of each variable by building buffers along edges of 30, 150, and 300 m widths, and then calculating between-site values within each buffer (Murphy et al., 2010). Since each of these metrics was highly correlated with the along-line calculations (*R*^2^ > 0.8), we used straight-line, 30 m width edge results for these metrics.

In spatially explicit genetic networks, incomplete sampling of nodes can lead to bias when using a pruned graph (Naujokaitis-Lewis et al., 2013). Given the small number of locations sampled for each species, we retained the fully connected networks for the gravity modeling procedure. Gravity models were run in R using the GeNetIt package. We used a hierarchical modeling approach to compare models that included one or more landscape variables with a distance-only (null) model. We used singly constrained models as they account for non-independence of pairwise comparisons. Gravity models were solved in mixed effects linear models using maximum likelihood (Zuur et al., 2009). We specified at-site and between-site variables as fixed effects and the identities of nodes as random effects (Murphy et al., 2010). We initially ran a null (distance) model and subsequently modeled at-site variables and between-site variables separately. We then built combined gravity models that included both classes of variables, via the inclusion of the best-supported, at-site and between-site variables identified during the first procedure. To avoid co-linearity, models did not include pairs of candidate variables correlated at 0.7 or higher (Table S5). We used Akaike information criterion scores adjusted for small sample size (AICc) to identify the best-supported models (Akaike, 1973; Burnham and Anderson, 2002). We additionally calculated conditional (including both fixed and random factors) *R*^2^ values for each model (Nakagawa and Schielzeth, 2013). We plotted network flow against each variable identified as significant in the best-supported models to assess the direction of the effects of candidate variables. We subsequently calculated cumulative AIC weight for each variable by summing the weights of each model in which this variable was included (Burnham and Anderson, 2002).

## Results

### Population graph analysis

**Figure 3:**
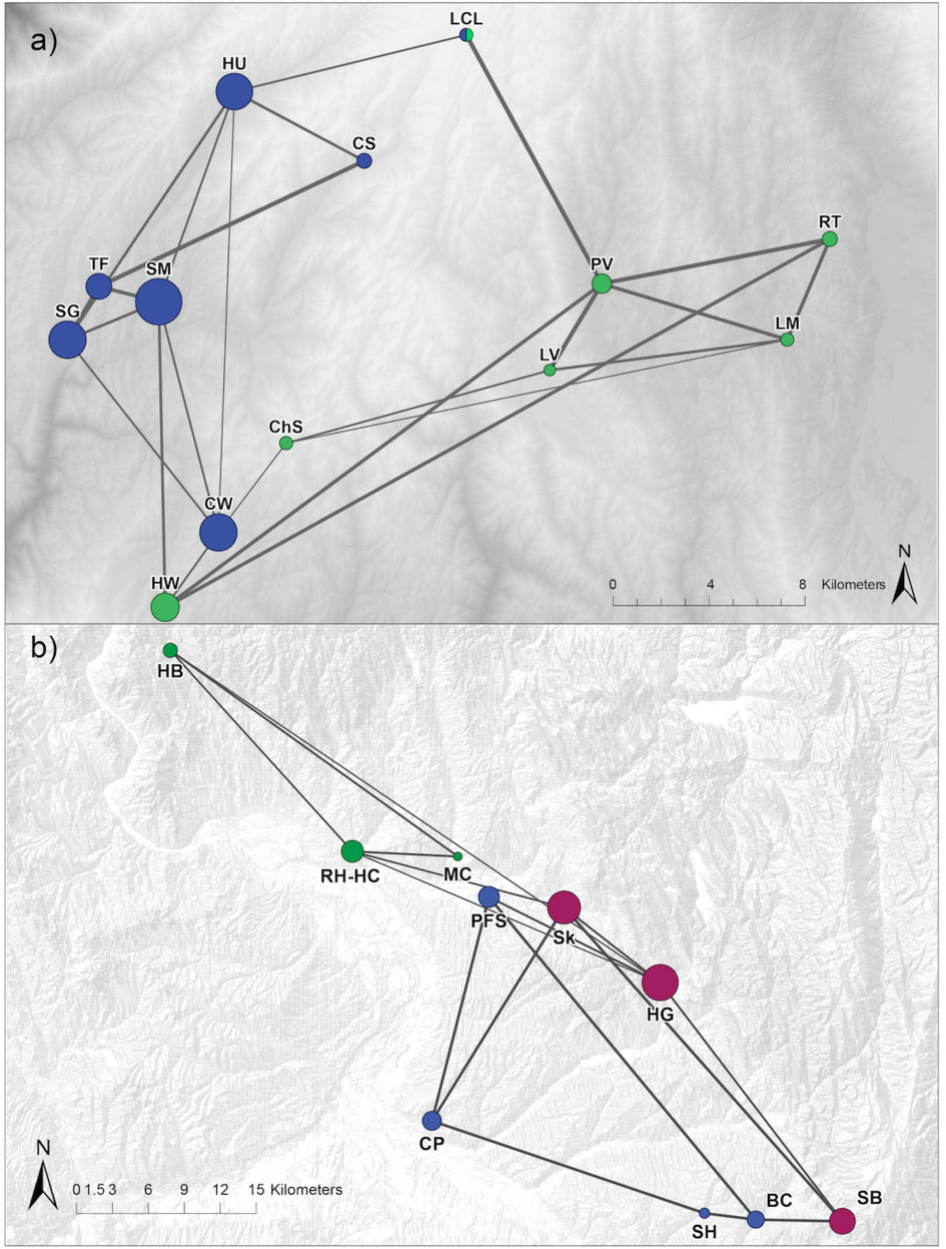
Network diagrams representing the genetic relationships between northern **(a)** and southern **(b)** Idaho ground squirrel sampling locations. Networks are pruned using conditional genetic distance (cGD; Dyer and Nason, 2004). Individuals were sampled during 2002–2006. Node colors differ by cluster assignment with the Girvan-Newman algorithm. Nodes are placed according to geographic location and scaled to reflect coreness, a network metric that quantifies proximity to the core in a core/periphery model (Table S3). Edge width is proportional to the genetic flow between sampling locations.

In the cGD pruning procedure, population graphs retained a total of 24 (31% of saturated network) edges connecting 13 nodes for NIDGS and 16 (36% of saturated network) edges connecting 10 nodes for SIDGS (Figure 3). We identified support for 2 and 3 genetic clusters via Girvan–Newman and Walktrap [modularity scores: Q(2)_Girvan−Newman_ = 0.33; Q(2)_Walktrap_ = 0.34, Q(3) = 0.33] for NIDGS. The 2-cluster model included 1 cluster in the northwestern portion of NIDGS range and a second cluster in the southeastern portion. Both algorithms agreed on all sampling location cluster assignments except study site LCL. For SIDGS, the model with 3 clusters received the highest modularity score [modularity scores: Q(2) = 0.26, Q(3)_Girvan−Newman_ = 0.29; Q(3)_Walktrap_ = 0.30]. Modularity, reflecting compartmentalization within each network, was slightly lower in SIDGS compared to NIDGS. The 3-cluster model included a cluster in the northwestern portion of the species' distribution, separated by the Weiser River and the agricultural area surrounding it from 2 discrete clusters, located in the southern and central area of the range. There was no evident spatial segregation between the southern and central clusters.

In NIDGS, the node strength centrality and betweenness metrics suggested higher connectivity for the western populations (Table S3). The coreness analysis provided the best support for a model with 5 nodes at the core and 8 at the periphery. For the 5-node core model, the concentration score was 0.91. The 5 core nodes, corresponding to the CW (betweenness = 15.08), HU (13.08), SG (2.25), SM (9.41), and HW (11.08) populations were located in the western portion of the range, confirming the patterns suggested by the other network metrics (Table S3). Spatial patterns were less evident in the network topology analysis of SIDGS. Sampling locations RH-HC (17.50), HG (29.44), SB (10.32), and CP (14.17), representing distinct areas of the species' range and all 3 genetic clusters, had the highest betweenness (Table S3). The core/periphery model results revealed that the optimal model included 3 nodes at the core (corresponding to HG, SB, and Sk) with a concentration score of 0.84.

**Figure 4:**
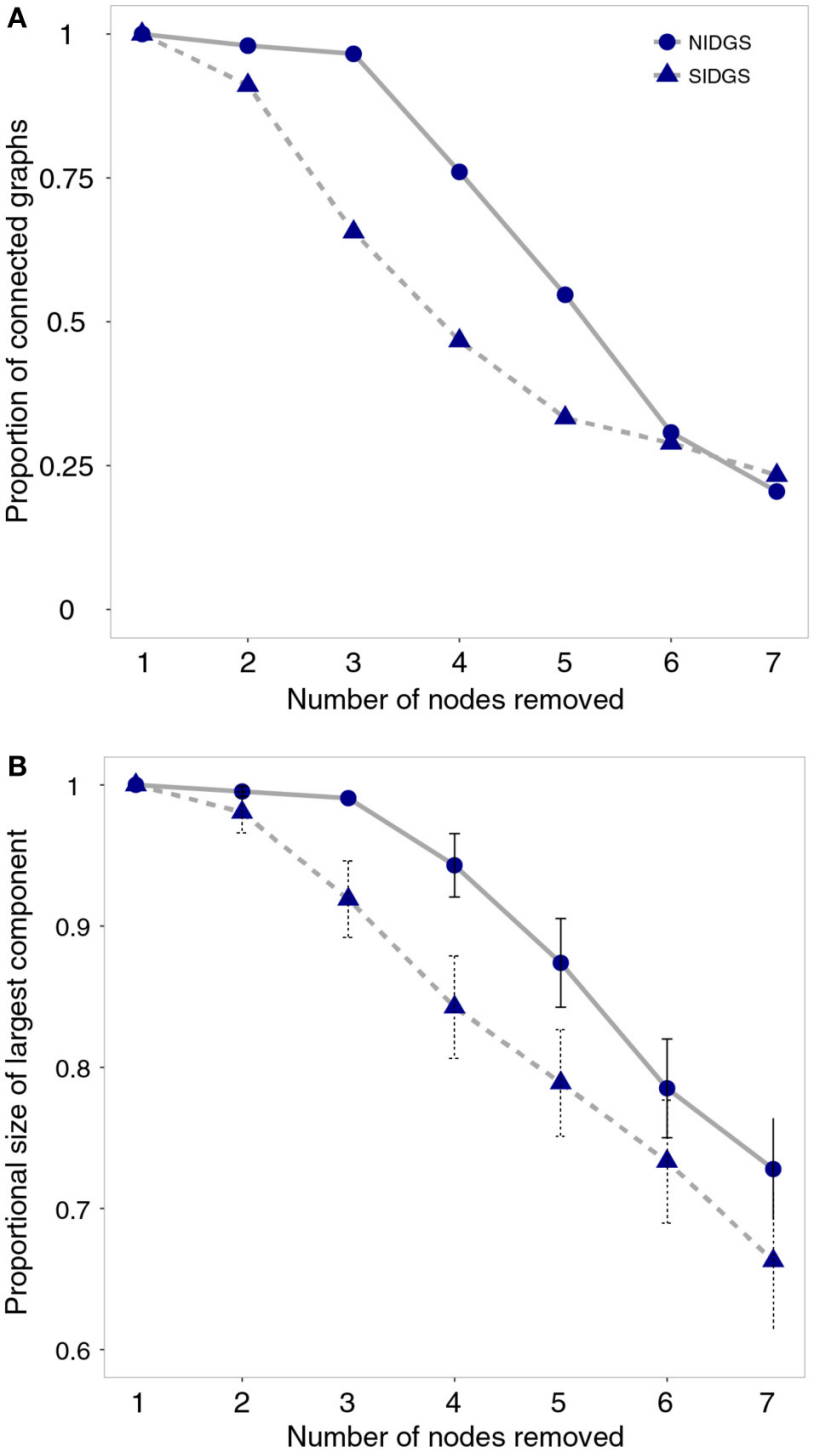
The effects of random sequential removal of nodes on proportion of connected graphs **(A)** and the largest remaining network component **(B)** for northern Idaho ground squirrels (solid line) and southern Idaho ground squirrels (dashed line). Proportions, means and 95% confidence intervals were calculated for up to 100 simulated networks in each scenario. Idaho ground squirrels were sampled from 23 locations during 2002–2006 and genotyped using 8 microsatellite loci (Hoisington-Lopez et al., 2012).

The node removal simulation analysis indicated that in the range of 2–5 removed nodes, SIDGS networks had higher probability of fragmentation by not creating a fully connected component (e.g., 3 nodes removed: NIDGS—97%, SIDGS—66% fully connected networks; Figure 4A). This larger fragmentation probability resulted in the largest components in SIDGS proportionally consisting of fewer nodes compared to NIDGS (e.g., 3 nodes removed: NIDGS—0.99, 95% CI = 0.98–1.0; SIDGS—92%, 95% CI = 0.9–0.94; Figure 4B). Taken together, both network connectivity metrics indicated higher resilience of the NIDGS network to node removal.

### Gravity models

The mean geographic distance between sampled locations for NIDGS and SIDGS was 16.1 and 28.9 km, respectively. For NIDGS subpopulations, the pairwise genetic distance (D_PS_) averaged (±SE) 0.41 ± 0.01, ranging from 0.23 to 0.56. SIDGS subpopulations had an average D_PS_ of 0.34 ± 0.02, ranging from 0.17 to 0.53. F_ST_ values were more similar among species, with means ±SE 0.19 ± 0.01 in NIDGS (range: 0.03–0.48) and 0.18 ± 0.01 for SIDGS (range: 0.04–0.41). For both species, pairwise genetic distance metrics were highly correlated (NIDGS *r* = 0.89; SIDGS *r* = 0.96). Mantel test results indicated a significant correlation between geographic distance and both metrics of genetic distance for NIDGS (D_PS_: *r* = 0.39, *P* = 0.001; F_ST_: *r* = 0.38, *P* = 0.002), and a stronger pattern in SIDGS (D_PS_: *r* = 0.64, *P* < 0.001; F_ST_: *r* = 0.57, *P* < 0.001).

**Figure 5:**
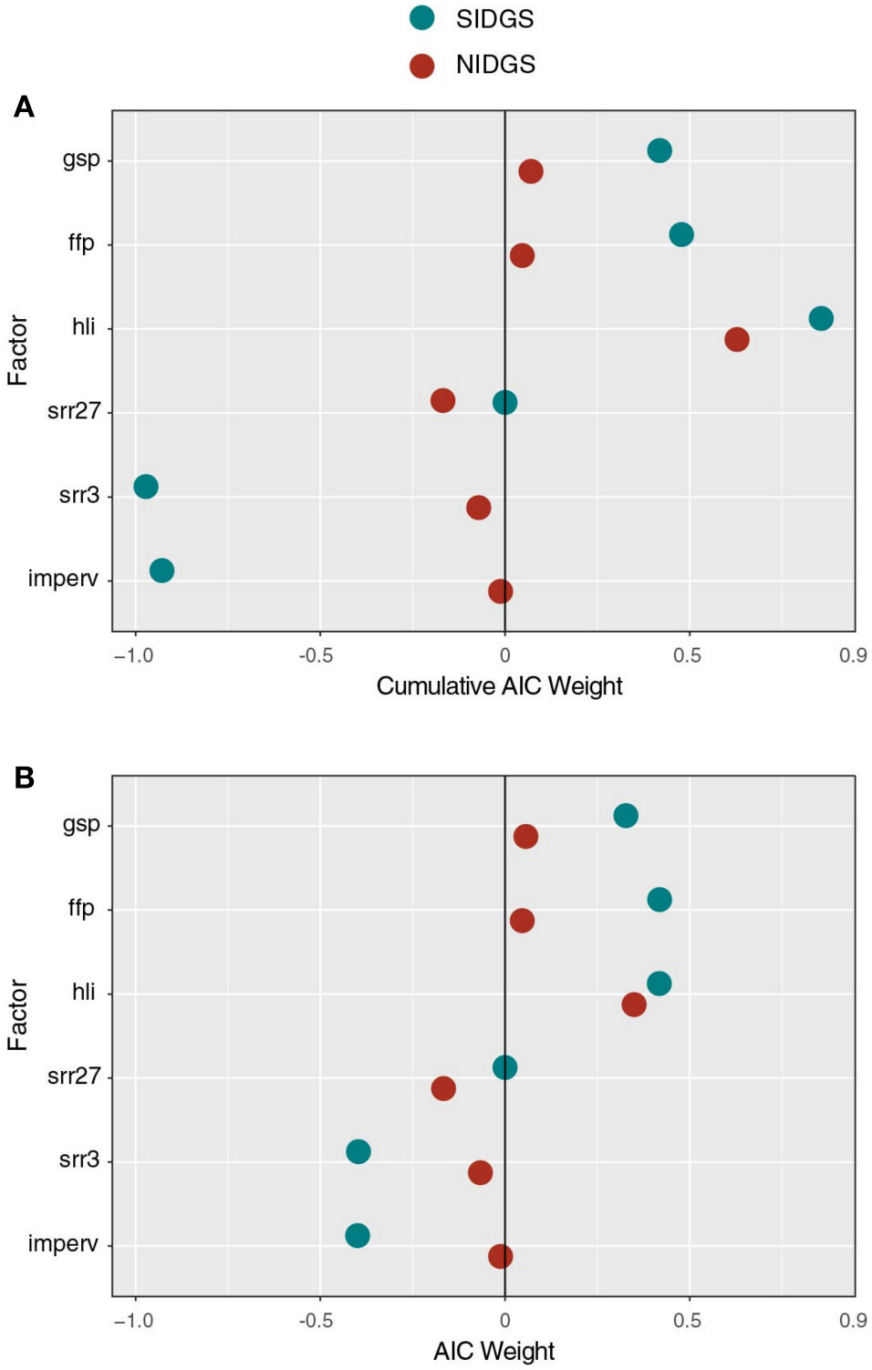
Cumulative AICc weights of several factors examined with gravity models as candidates for driving NIDGS (red circles) and SIDGS (blue circles) gene flow. Panels include cumulative AICc weights for each factor in all examined models **(A)** and only the top-ranked model for each factor **(B)**. Positive and negative values represent the direction and magnitude of relative support for each factor's influence.

The top variables for the NIDGS saturated network included those associated with potential site productivity (*v*: *hli, gsp*) and topography (*c*: *srr27, srr3*; Table 2, Figure 5). For NIDGS, geographic distance (*w*) was the sixth-ranked model, with a ΔAICc of 4.4. Heat load index (*hli*) positively correlated with gene flow and had the greatest support among at-site variables (variable weight: 0.63), while growing season precipitation had a weight of 0.06. One additional at-site variable, frost-free period (*ffp*: 0.04), received some variable weight but did not appear in those models that improved on the distance-only model. Measures of large-scale (*srr27*: 0.54) and small-scale (*srr3*: 0.08) topographic complexity negatively correlated to gene flow, and were the between-site variables with the greatest weights. Variables describing land cover, interspecific competition, and human disturbance received negligible support (Table S3). For individual parameter estimates, see Table S6.

**Table 2:** Gravity model results of the best-supported models for northern and southern Idaho ground squirrels.

**Species**	**Full model description**	**Type**	**ΔAICc**	**AIC weight**	**Conditional *R*^2^**
northern Idaho ground squirrel	*w + hli − srr27*	at + between	0.0	0.33	0.40
	*w − srr27*	between	1.3	0.17	0.41
	*w + hli*	at	2.5	0.09	0.36
	*w + hli − srr3*	at + between	3.9	0.05	0.37
	*w + gsp + hli − srr27*	at + between	4.2	0.04	0.39
	*w*	distance	4.4	0.04	0.38
southern Idaho ground squirrel	*w + ffp + hli − imperv − srr3*	at + between	0	0.42	0.47
	*w + gsp + hli − imperv − srr3*	at + between between	0.5	0.33	0.47
	*w + gsp − imperv − srr3*	at + between between	3.6	0.07	0.46
	*w + hli − imperv − srr3*	at + between	3.9	0.06	0.44
	*w + ffp − imperv − srr3*	at + between	5.3	0.03	0.46
	*w + ffp + hli − agri − srr3*	at + between	6.7	0.01	0.44
	*w − imperv − srr3*	between	6.9	0.01	0.44
	*w + gsp + hli − agri − srr3*	at + between	7.3	0.01	0.44
	*w*	distance	15	0.00	0.37

*Type indicates whether the model includes at-site, between-site, or both categories of predictors. A full list of models in available in Tables S3, S4*.

For SIDGS, gene flow was positively correlated with at-site productivity and negatively correlated with between-site factors associated with reduced landscape permeability including human disturbance (*imperv, agri*) and small-scale topographic complexity (*srr3*; Table 2). For SIDGS, heat load index (*hli*) at sites was positively correlated with gene flow (variable weight: 0.83). Growing season precipitation (*gsp*: 0.42) and frost-free period (*ffp*: 0.46) also positively related to gene flow. Small-scale topographic complexity (*srr3*: 0.95) appeared in all top models and negatively correlated with gene flow. Impervious surfaces appeared in six of eight top models, contributing 92% AIC weight, and was negatively correlated with gene flow. Agricultural areas impeded gene flow, but this land cover type received minimal weight (*agri*: 0.02). Variables describing land cover classes and stream densities received negligible support (Table S4).

## Discussion

We combined two graph theoretic approaches to enhance our understanding of the functional connectivity of two Idaho ground squirrel species and to inform conservation efforts. Population graph analysis revealed that the pattern and strength of network connectedness differed by species. Node removal simulations suggested that in the event of local patch extinction, SIDGS would likely lose connectivity rapidly, while NIDGS would maintain gene flow despite the removal of several patches or nodes. Gravity models revealed the influence of at-site productivity variables in both species, a finding that would not have been detected in traditional network approaches. These models also revealed effects of topographic complexity at two different spatial scales: fine-scale variation for SIDGS and broad-scale and fine-scale variation for NIDGS. Development, as measured by impervious surfaces, was a major hindrance to SIDGS gene flow.

### Patterns of genetic structure

We found support for 2 or 3 genetic clusters in NIDGS and similar support for 3 genetic clusters in SIDGS using network community detection (Newman, 2006). Functional connectivity among habitat patches in NIDGS appears to be limited by a mountain ridge, with subpopulations clustered in the northwestern and southeastern portions of the range, and this result is similar to that obtained via STRUCTURE (Pritchard et al., 2000; Hoisington, 2007). However, one site (CW) located in the southwestern portion of the species' range (Figure 3), deviated from this pattern. Interestingly, the population graph links CW to 3 populations in the northwestern cluster, and the 2 populations spatially adjacent to CW (ChS and HW) in the southeastern genetic cluster. This pattern, which is consistent with mitochondrial DNA analyses (Hoisington, 2007), could be explained by repeated translocations of individuals from SM and surrounding sites into CW (Gavin et al., 1999) as well as natural recolonization from HW. For SIDGS, our population graph detected a unique genetic cluster in the northern portion of its range, composed of 3 populations. This suggests that the Weiser River acts as a barrier to gene flow as suggested in previous analyses (Garner et al., 2005; Hoisington, 2007). The optimal model for SIDGS had a modularity score lower than 0.3. Our results reveal genetic connectivity across the southern portion of the SIDGS range despite considerable fragmentation due to agriculture. In general, there was congruence between the community detection results and previous Bayesian clustering analyses (Hoisington, 2007). Population graph community detection algorithms base their calculations on genetic distances among nodes and thus have the advantage of including the contribution of ancestries from other genetic clusters. The similarity in results suggests that these methods are well suited for our study system and applicable in additional systems where genetic data can be represented as population graphs.

Network topology metrics, calculated at the sampling location level, were consistent with these patterns. NIDGS nodes with the highest strength and betweenness were the ones that belonged to the core according to the core-periphery model. One exception was PV, which is spatially central and highly connected, but did not constitute a core node. All NIDGS core nodes were found in the western portion of the species' range. In addition, the majority of edges among these nodes were retained in the pruned network (Figure 3A). This may indicate that the western portion of the range is a source for dispersing individuals. With the exception of the CW population, the NIDGS population graph topology indicates a west-to-east gradient of connectivity. In SIDGS, nodes with high overall connectivity according to degree, strength, and betweenness, such as CP and PFS, were not included in the core and had relatively lower coreness (Figure 3B; Table S3). Core nodes (HG, SB, and Sk) all belonged to the same genetic cluster and were located in the southeastern part of the species' range. Interestingly, the most spatially central locations (PFS and MC) were not the most connected ones, suggesting that there are additional factors driving SIDGS gene flow beyond geographic distance.

The correlation of network structure to the idealized core/periphery model was slightly higher for NIDGS (0.91) compared to SIDGS (0.84), as was the proportion of core nodes. This slight difference may be explained by the lack of spatial organization in the SIDGS network (Figure 3). Overall, our population graph analyses indicate that gene flow among NIDGS locations is higher compared to SIDGS, which is consistent with the relatively large geographic distances found among SIDGS populations. Our use of core/periphery models to assess genetic data is a novel application of a methodology previously developed for social networks (Borgatti and Everett, 1999), and provides an additional metric to quantify node contribution, which may reflect the degree to which discrete sites are sources or sinks for dispersers.

Simulated node removals indicated an immediate decline in overall connectivity among SIDGS nodes, compared to the relative robustness to node removal in NIDGS (Figure 4), suggesting that the few connections retained in the SIDGS population graph have an increased conservation value for this species. In addition, these results imply that local extinction of 2 current subpopulations would drive a substantial decline in functional connectivity. SIDGS occur in areas prone to intense human activity and subpopulations are separated by large geographic distances. Our results highlight the susceptibility of this species to future habitat loss and fragmentation, and raise concern over further isolation of the remaining subpopulations. In contrast, simulated node removal in the NIDGS population graph suggests that this species is relatively robust to localized extinctions. The pruned population graph retained a similar proportion of edges, comprised of shorter distances, among subpopulations compared to the SIDGS graph. These results, in conjunction with lower levels of human disturbance across the NIDGS range, suggest that in the event of local extinctions the species may be better able to maintain population connectivity (Fahrig, 2002; Driscoll, 2004).

### Functional connectivity

The variables that were important in gravity models differed between species. We predicted that at-site variables associated with potential productivity would be positively correlated with functional connectivity for both species. Population size at each site would likely be an important predictor of gene flow, but these data were not available. However, population estimates are relevant to the conservation of both species and should be a priority for data collection. We also hypothesized that between-site variables indicative of high habitat quality would facilitate gene flow, while variables reflecting human activity would inhibit gene flow.

Model fit, as measured by conditional R^2^, was moderate for both species (*R*^2^ ~ 0.4). These results could be an artifact of our limited power to detect variation in habitat variables across the study areas (Short Bull et al., 2011), especially in light of the small number of extant populations occurring over restricted ranges (Figure 1).

Nevertheless, a number of at-site variables were identified as predictors of gene flow. Heat load index (*hli*), a surrogate for vegetation productivity, was one at-site variable that contributed to gene flow in both species. This metric had substantial cumulative AIC weight across models of NIDGS (*w* = 0.63) and SIDGS (*w* = 0.83) connectivity. Sites with a higher *hli* may yield a larger number of squirrels with improved body conditions due to increased forage availability and quality. The finding that NIDGS are primarily structured, apart from isolation by distance, by at-site productivity, would have been difficult to detect with other landscape genetic statistical approaches. Additionally, two other at-site variables associated with potential productivity facilitated gene flow for both species. Longer frost-free periods and increased growing-season precipitation were associated with higher connectivity. Lohr et al. (2013) reported that the greatest densities of SIDGS were associated with higher cover of perennial grasses, native perennial forbs, and higher plant species diversity. The combination of solar intercept (*hli*), long growing season (*ffp*), and greater rainfall (*gsp*) may result in high forage quality and quantity for ground squirrels. Therefore, at-site vegetation production is likely an important characteristic in maintaining viable populations for both species.

Landscape features that restricted gene flow differed for the two species. The population graph results for NIDGS revealed a division between the western and eastern sampling areas that are geographically separated by a mountain ridge. This is mirrored in the gravity model results, for which large-scale topographic complexity (*srr27*) received 54% weight across models. At this broad scale, *srr* is likely detecting ridges as a filter to movement, and this pattern is visually apparent when the graph of population structure is overlaid on topography (Figure 3A). Three landscape features were identified as barriers to gene flow for SIDGS: impervious surfaces, small-scale topographic complexity, and, to a minor extent, agriculture. Populations were less connected in highly developed areas as measured by imperviousness of surfaces along edges connecting nodes. Impervious surfaces primarily reflect the presence of roads. Gene flow could be disrupted across roads due to avoidance of high traffic areas or altered roadside habitat, increased mortality from vehicle collisions, or a combination of these factors. Although roads are often considered an important source of mortality for many wildlife species (Forman, 1998), small mammals may select these areas (Oxley et al., 1974), and the effects on small mammal behavior and movement may be contingent on road type and traffic volume (Brock and Kelt, 2004). Previous results indicate that dispersing Idaho ground squirrels repeatedly use dirt roads as corridors (Panek, 2005). The absence of support for road effects on NIDGS could be attributed to lower densities of high-volume traffic (paved) roads surrounding the sampling sites for this species. The negative impact of agricultural areas on gene flow may imply an avoidance of these areas, although the variable weight for this metric was small.

Restriction of gene flow in both species due to small-scale topographic complexity (*srr3*) likely reflects a preference for low-elevation, flat grasslands characteristic of the meadows. Gravity models failed to show any support for either ephemeral or perennial streams as drivers of gene flow (Tables S3, S4). However, our population graph analysis identified the Weiser River as a likely barrier to gene flow. Thus, our inability to detect an important barrier to gene flow with gravity models was supplemented by the results from our population graph analysis. These complementary results highlight the benefits of using multiple analytical methods for detecting patterns in genetic data.

### Conservation implications

Our findings of differences in functional connectivity and its drivers highlight the need for different conservation and management strategies for each species of Idaho ground squirrel. Results from the node removal analysis suggest that NIDGS populations are more connected and relatively resistant to metapopulation collapse from local population extinctions. Although, SIDGS are no longer a candidate for federal listing, their subpopulations may be more susceptible to future habitat loss and fragmentation than NIDGS (Hoisington-Lopez et al., 2012). Connectivity in NIDGS was driven mainly by potential site productivity and topographic characteristics, and not a lack of suitable habitat. These combined lines of evidence suggest that recent conservation efforts for NIDGS have been effective at maintaining this species' gene flow and diversity, and should therefore be continued.

Our results for southern Idaho ground squirrels suggest this species is extremely vulnerable. SIDGS sites are geographically distant from one another and highly sensitive to node removal (i.e., local extinction). Sites that are poorly connected, and thus unlikely to be recolonized following an extirpation event, may be good candidates for reintroduction. Additionally, sites that are highly connected might be examined for landscape characteristics that could be used as part of novel site reintroduction selection criteria. Translocations have been attempted with apparent success for SIDGS (Yensen and Tarifa, 2012), and these efforts, combined with supplementation from captive breeding, may become important for maintaining genetic connectivity and diversity in SIDGS populations (Hoisington-Lopez et al., 2012). Given the distances that separate SIDGS sites, we support the recommendation of Garner et al. (2005) that managers consider establishing additional populations to serve as stepping stones for connectivity. Our gravity model results suggest that factors relating to at-site vegetation productivity affect SIDGS genetic structure. A large amount of SIDGS habitat is located either in agricultural areas or sites dominated by invasive cheatgrass, both of which may be difficult to restore. While it appears that NIDGS have responded positively to habitat restoration, this strategy is less likely to successfully improve SIDGS habitat and functional connectivity due to the pervasive invasion of exotic weeds in their range (Yensen, 1991).

## Conclusions

When working with species of conservation concern, it is important not only to assess genetic structure, but also to identify the factors that influence genetic connectivity. Here, we illustrate the value of using recently developed network-based approaches to examine functional connectivity for two vulnerable species of Idaho ground squirrels. Population graphs enhanced our understanding of each species' resistance to potential future loss of habitat patches or populations. Gravity models provided new insights into landscape-related processes that drive genetic structure of these imperiled species, particularly by identifying at-site influences on gene flow. We conclude that the combination of these methodologies allows stronger inference and a more complete assessment of genetic structure. Network models are especially advantageous for representing gene flow in species exhibiting patchy distributions. We encourage further exploration of these methodologies as a framework for hypothesis testing in future landscape genetics studies.

## Author contributions

MM and LW conceived the research. JH collected data. VZ, AB, DJ, AP, XG, DT, RP, and JH analyzed data. All authors contributed to the writing of the manuscript.

### Conflict of interest statement

The authors declare that the research was conducted in the absence of any commercial or financial relationships that could be construed as a potential conflict of interest.
