# Supplementary material for: Complementary Network-Based Approaches for Exploring Genetic Structure and Functional Connectivity in Two Vulnerable, Endemic Ground Squirrels
Source: Front Genet. 2017 Jun 14;8:81. doi: 10.3389/fgene.2017.00081 (PMC5469978; doi:10.3389/fgene.2017.00081)
Supplement: Supplementary file 1 [file Table1.DOCX]

Supplement: Complementary network-based approaches reveal conservation priorities for two vulnerable ground squirrel species

Victoria H. Zero^*^, Adi Barocas^*^, Denim M. Jochimsen, Agnes Pelletier, Xavier Giroux-Bougard, Daryl R. Trumbo, Jessica Castillo, Diane Evans Mack, Mark A. Linnell, Rachel M. Pigg, Jessica Hoisington-Lopez, Stephen F. Spear, Melanie A. Murphy, Lisette P. Waits

*These authors contributed equally to this work

**Table S1.** Sample sizes for **(A)** northern Idaho ground squirrel and **(B)** southern Idaho ground squirrel.

| **Sampling area** | **Abb.** | **Total** | **Females** | **Males** |
| --- | --- | --- | --- | --- |
| **A)** |  |  |  |  |
| Chipmunk Springs | ChS | 18 | 16 | 2 |
| Cold Springs | CS | 35 | 21 | 14 |
| Cottonwood | CW | 6 | 3 | 3 |
| Huckleberry | HU | 21 | 13 | 8 |
| Halfway | HW | 17 | 11 | 6 |
| Lick Creek Lookout | LCL | 18 | 15 | 3 |
| Little Mud | LM | 13 | 10 | 3 |
| Lost Valley | LV | 29 | 17 | 12 |
| Price Valley | PV | 28 | 15 | 13 |
| Ridgetop | RT | 13 | 10 | 3 |
| Summit Gulch | SG | 35 | 28 | 7 |
| Squirrel Manor | SM | 18 | 15 | 3 |
| Tree Farm | TF | 20 | 10 | 10 |
| Average |  | 21.0 | 14.4 | 6.6 |
| **B)** |  |  |  |  |
| Bissel Creek | BC | 65 | 32 | 33 |
| Clay Peak | CP | 24 | 12 | 12 |
| Henley Basin | HB | 12 | 9 | 3 |
| Holland Gulch | HG | 10 | 7 | 3 |
| Mann Creek | MC | 20 | 7 | 13 |
| Phillip Farm South | PFS | 22 | 12 | 10 |
| Hillcrest Cemetery/ Rolling hills | HC- RH | 66 | 42 | 24 |
| Squaw Butte | SB | 53 | 47 | 6 |
| Sand Hollow | SH | 21 | 13 | 8 |
| Skow | Sk | 19 | 16 | 3 |
| Average |  | 28.4 | 17.9 | 10.5 |

**Table S2.** Network metrics calculated for each sampling area for northern Idaho ground squirrels (a) and southern Idaho ground squirrels (b) sampled during 2002-2006 and genotyped using 8 microsatellite loci (Hoisington-Lopez et al. 2012). The population graph used was based on conditional genetic distance (cGD). Degree is the number connections associated with each node; Strength centrality is the extension of degree for valued networks; Betweenness centrality quantifies the number of shortest paths that a particular node or edge lies on and indicates the importance of a node in terms of the bottleneck it creates; Coreness represents the node’s adjacency to the core in a continuous core/periphery model. Asterisks denote nodes which belong to the core.

| Sampling area | Abb | Degree | Strength | Betweenness | Coreness |
| --- | --- | --- | --- | --- | --- |
| a) |  |  |  |  |  |
| Chipmunk Springs | ChS | 3 | 2.00 | 8.0 | 0.13 |
| Cold Springs | CS | 2 | 1.54 | 0.67 | 0.15 |
| Cottonwood | CW | 5 | 3.33 | 15.08 | 0.40* |
| Huckleberry | HU | 5 | 3.53 | 13.08 | 0.38* |
| Halfway | HW | 4 | 2.97 | 11.08 | 0.29* |
| Lick Creek Lookout | LCL | 2 | 1.51 | 4.91 | 0.13 |
| Little Mud | LM | 4 | 2.93 | 2.33 | 0.13 |
| Lost Valley | LV | 3 | 2.26 | 0.83 | 0.10 |
| Price Valley | PV | 5 | 3.98 | 10.67 | 0.20 |
| Ridgetop | RT | 3 | 2.37 | 1.08 | 0.15 |
| Summit Gulch | SG | 4 | 2.96 | 2.25 | 0.40* |
| Squirrel Manor | SM | 5 | 3.65 | 9.41 | 0.49* |
| Tree Farm | TF | 4 | 2.38 | 1.58 | 0.26 |
| b) |  |  |  |  |  |
| Bissel Creek | BC | 3 | 2.57 | 8.06 | 0.25 |
| Clay Peak | CP | 3 | 2.48 | 14.17 | 0.27 |
| Henley Basin | HB | 3 | 1.97 | 4.82 | 0.20 |
| Hillcrest Cemetery/ Rolling Hills | HC-RH | 4 | 2.65 | 17.50 | 0.31 |
| Holland Gulch | HG | 5 | 3.39 | 29.44 | 0.51* |
| Mann Creek | MC | 2 | 1.44 | 0.00 | 0.12 |
| Phillips Farm South | PFS | 3 | 2.44 | 11.02 | 0.30 |
| Squaw Butte | SB | 3 | 2.38 | 10.32 | 0.36* |
| Sand Hollow | SH | 2 | 1.68 | 1.39 | 0.15 |
| Skow | Sk | 4 | 3.02 | 22.73 | 0.46* |

**Table S3.** Model structure, relative support based on Akaike Information Criterion corrected for small sample size (AICc), and conditional R^2^  (Nakagawa and Schielzeth 2013) of candidate gravity models for northern Idaho ground squirrel (NIDGS) based on a saturated 1 – Dps (flow) network. Coding for model descriptions follow table 1 in main text. NIDGS samples include 316 individuals from 13 populations in central Idaho, collected between 2004 and 2006 and genotyped at 8 microsatellite loci.

| **Full model description** | **Type** | **K** | **logLike** | **AICc** | **ΔAICc** | **AIC weight** | **conditional R^2^** |
| --- | --- | --- | --- | --- | --- | --- | --- |
| *w + hli - SRR27* | at + between | 3 | 76.36 | -144.04 | 0.0 | 0.33 | 0.39 |
| *w - SRR27* | between | 2 | 73.99 | -142.78 | 1.3 | 0.17 | 0.41 |
| *w + hli* | at | 2 | 73.37 | -141.53 | 2.5 | 0.09 | 0.36 |
| *w + hli - SRR3* | at + between | 3 | 74.39 | -140.10 | 3.9 | 0.05 | 0.37 |
| *w + gsp + hli - SRR27* | at + between | 4 | 76.41 | -139.81 | 4.2 | 0.04 | 0.39 |
| *w* | between | 1 | 71.02 | -139.67 | 4.4 | 0.04 | 0.38 |
| *w - SRR3* | between | 2 | 72.07 | -138.94 | 5.1 | 0.03 | 0.38 |
| *w + hli + ffp* | at | 3 | 73.77 | -138.86 | 5.2 | 0.02 | 0.36 |
| *w + ffp* | at | 2 | 71.93 | -138.66 | 5.4 | 0.02 | 0.37 |
| *w + hli + shrub* | at + between | 3 | 73.64 | -138.61 | 5.4 | 0.02 | 0.36 |
| *w + hli + gsp* | at | 3 | 73.63 | -138.59 | 5.5 | 0.02 | 0.36 |
| *w + hli + cogs* | at | 3 | 73.61 | -138.56 | 5.5 | 0.02 | 0.36 |
| *w + hli + grass* | at | 3 | 73.44 | -138.21 | 5.8 | 0.02 | 0.36 |
| *w + hli + grass* | at + between | 3 | 73.38 | -138.09 | 6.0 | 0.02 | 0.36 |
| *w - eph_strm* | between | 2 | 71.56 | -137.93 | 6.1 | 0.02 | 0.38 |
| *w + shrub* | between | 2 | 71.47 | -137.74 | 6.3 | 0.01 | 0.37 |
| *w - perm_strm* | between | 2 | 71.44 | -137.67 | 6.4 | 0.01 | 0.39 |
| *w - imperv* | between | 2 | 71.29 | -137.38 | 6.7 | 0.01 | 0.38 |
| *w - agri* | between | 2 | 71.17 | -137.14 | 6.9 | 0.01 | 0.39 |
| *w + gsp* | at | 2 | 71.05 | -136.91 | 7.1 | 0.01 | 0.38 |
| *w + grass* | at | 2 | 71.05 | -136.90 | 7.1 | 0.01 | 0.38 |
| *w + cogs* | at | 2 | 71.02 | -136.83 | 7.2 | 0.01 | 0.38 |
| *w + grass* | between | 2 | 71.02 | -136.83 | 7.2 | 0.01 | 0.38 |
| *w + shrub - eph_strm* | between | 3 | 72.06 | -135.45 | 8.6 | 0.00 | 0.41 |
| *w + shrub - imperv* | between | 3 | 72.06 | -135.45 | 8.6 | 0.00 | 0.37 |
| *w + hli + shrub+grass* | at + between | 4 | 73.70 | -134.40 | 9.6 | 0.00 | 0.36 |
| *w + hli + ffp + shrub* | at + between | 4 | 72.15 | -131.30 | 12.7 | 0.00 | 0.36 |

**Table S4.** Model structure, relative support based on Akaike Information Criterion corrected for small sample size (AICc), and conditional R^2^  (Nakagawa and Schielzeth 2013) of candidate gravity models for southern Idaho ground squirrel (SIDGS) based on a saturated 1 – Dps (flow) network. Coding for model descriptions follow Table 1 in the main text. SIDGS samples include 263 individuals from 10 populations in central Idaho, collected between 2002 and 2006 and genotyped at 8 microsatellite loci.

| **Full model description** | **Type** | **K** | **logLike** | **AICc** | **ΔAICc** | **AIC weight** | **conditional R^2^** |
| --- | --- | --- | --- | --- | --- | --- | --- |
| *w + ffp + hli - imperv - err3* | at + between | 6 | 8.3 | -0.6 | 0 | 0.42 | 0.47 |
| *w + gsp + hli - imperv - err3* | at + between | 6 | 8.1 | -0.1 | 0.5 | 0.33 | 0.47 |
| *w + gsp - imperv - err3* | at + between | 5 | 5.5 | 3 | 3.6 | 0.07 | 0.46 |
| *w + hli - imperv - err3* | at + between | 5 | 5.4 | 3.3 | 3.9 | 0.06 | 0.44 |
| *w + ffp - imperv - err3* | at + between | 5 | 4.6 | 4.7 | 5.3 | 0.03 | 0.46 |
| *w + ffp + hli - agri - err3* | at + between | 6 | 4.9 | 6.1 | 6.7 | 0.01 | 0.44 |
| *w + - imperv - err3* | between | 4 | 2.9 | 6.3 | 6.9 | 0.01 | 0.44 |
| *w + gsp + hli - agri - err3* | at + between | 6 | 4.7 | 6.7 | 7.3 | 0.01 | 0.44 |
| *w + gsp - agri - err3* | at + between | 5 | 2.9 | 8.2 | 8.8 | 0.01 | 0.44 |
| *w + hli - agri - err3* | at + between | 5 | 2.9 | 8.3 | 8.9 | 0.00 | 0.43 |
| *w + err3* | between | 3 | 0.6 | 8.8 | 9.4 | 0.00 | 0.42 |
| *w + ffp - agri - err3* | at + between | 5 | 2.3 | 9.3 | 9.9 | 0.00 | 0.45 |
| *w + grass - err3* | between | 4 | 1.2 | 9.5 | 10.1 | 0.00 | 0.43 |
| *w + ffp + hli - agri* | at + between | 5 | 2.3 | 9.5 | 10.1 | 0.00 | 0.41 |
| *w + ffp + hli* | at | 4 | 1 | 10 | 10.6 | 0.00 | 0.38 |
| *w + gsp + hli - agri* | at + between | 5 | 2 | 10 | 10.6 | 0.00 | 0.41 |
| *w - imperv + shrub* | between | 4 | 1 | 10.1 | 10.7 | 0.00 | 0.40 |
| *w + - agri - err3* | between | 4 | 0.9 | 10.2 | 10.8 | 0.00 | 0.43 |
| *w + - shrub - err3* | between | 4 | 0.7 | 10.5 | 11.1 | 0.00 | 0.42 |
| *w + gsp + hli* | at | 4 | 0.7 | 10.7 | 11.3 | 0.00 | 0.38 |
| *w + shrub* | between | 3 | -0.4 | 10.8 | 11.4 | 0.00 | 0.40 |
| *w + hli - agri* | at + between | 4 | 0.5 | 10.9 | 11.5 | 0.00 | 0.40 |
| *w + ffp + hli - imperv* | at + between | 5 | 1.2 | 11.6 | 12.2 | 0.00 | 0.38 |
| *w + hli* | at | 3 | -1.1 | 12.1 | 12.7 | 0.00 | 0.37 |
| *w + shrub + hli* | at | 4 | 0 | 12.1 | 12.7 | 0.00 | 0.38 |
| *w + soil* | at | 3 | -1.1 | 12.2 | 12.8 | 0.00 | 0.38 |
| *w + gsp + hli - imperv* | at + between | 5 | 0.8 | 12.4 | 13 | 0.00 | 0.38 |
| *w + gsp* | at | 3 | -1.3 | 12.5 | 13.1 | 0.00 | 0.39 |
| *w + shrub - agri* | between | 4 | -0.3 | 12.6 | 13.2 | 0.00 | 0.40 |
| *w - agri* | between | 3 | -1.4 | 12.7 | 13.3 | 0.00 | 0.39 |
| *w + shrub + grass* | between | 4 | -0.4 | 12.8 | 13.4 | 0.00 | 0.40 |
| *w + ffp* | at | 3 | -1.8 | 13.6 | 14.2 | 0.00 | 0.39 |
| *w - perm.strm* | between | 3 | -1.8 | 13.7 | 14.3 | 0.00 | 0.39 |
| *w + hli - imperv* | at + between | 4 | -1 | 14.1 | 14.7 | 0.00 | 0.37 |
| *w* | distance | 2 | -3.2 | 14.4 | 15 | 0.00 | 0.37 |
| *w + gsp - imperv* | at + between | 4 | -1.2 | 14.5 | 15.1 | 0.00 | 0.39 |
| *w + grass - agri* | between | 4 | -1.3 | 14.6 | 15.2 | 0.00 | 0.39 |
| *w - err27* | between | 3 | -2.4 | 14.9 | 15.5 | 0.00 | 0.38 |
| *w + shrub + ffp* | at | 4 | -1.6 | 15.2 | 15.8 | 0.00 | 0.39 |
| *w + ffp - imperv* | at + between | 4 | -1.8 | 15.6 | 16.2 | 0.00 | 0.39 |
| *w + eph.strm* | between | 3 | -2.9 | 15.8 | 16.4 | 0.00 | 0.37 |
| *w + shrub* | at | 3 | -3 | 16 | 16.6 | 0.00 | 0.37 |
| *w + grass* | at | 3 | -3 | 16 | 16.6 | 0.00 | 0.37 |
| *w + grass* | between | 3 | -3 | 16 | 16.6 | 0.00 | 0.37 |
| *w - imperv* | between | 3 | -3.2 | 16.4 | 17 | 0.00 | 0.37 |
| *w - imperv + grass* | between | 4 | -2.9 | 17.9 | 18.5 | 0.00 | 0.37 |

**Table S5.** Spearman correlation coefficients among at-site and between-site variables used for gravity modeling in NIDGS and NIDGS. Description of variables is provided in Table 1.

| NIDGS | *gsp* | | *grass* | | *hli* | | *cogs* | SIDGS | *gsp* | *ffp* | *hli* | *grass* | *shrub* |
| --- | --- | --- | --- | --- | --- | --- | --- | --- | --- | --- | --- | --- | --- |
| *ffp* | -0.53 | | -0.07 | | -0.33 | | -0.20 | *Soil* | -0.47 | 0.33 | 0.46 | -0.21 | -0.18 |
| *gsp* |  | | 0.00 | | 0.18 | | -0.07 | *gsp* |  | -0.88 | -0.34 | -0.10 | 0.70 |
| *grass* |  | |  | | -0.38 | | 0.05 | *ffp* |  |  | -0.04 | 0.02 | -0.84 |
| *hli* |  | |  | |  | | 0.42 | *hli* |  |  |  | 0.02 | 0.20 |
|  | |  |  |  | |  | | *grass* |  |  |  |  | -0.31 |

| NIDGS | *srr3* | *shrub* | *per_strm* | *imperv* | *grass* | *eph_strm* | *agri* |
| --- | --- | --- | --- | --- | --- | --- | --- |
| *srr27* | 0.73 | 0.08 | 0.52 | 0.16 | -0.14 | 0.00 | 0.15 |
| *srr3* |  | 0.06 | 0.48 | 0.00 | -0.08 | -0.03 | 0.13 |
| *shrub* |  |  | 0.03 | 0.13 | 0.13 | 0.18 | 0.37 |
| *per_strm* |  |  |  | 0.15 | -0.45 | -0.21 | -0.05 |
| *imperv* |  |  |  |  | -0.12 | 0.22 | -0.08 |
| *grass* |  |  |  |  |  | 0.19 | 0.17 |
| *eph_strm* |  |  |  |  |  |  | 0.05 |

| SIDGS | *imperv* | *agri* | *grass* | *shrub* | *per_strm* | *srr3* | *srr27* |
| --- | --- | --- | --- | --- | --- | --- | --- |
| *eph_strm* | -0.32 | -0.75 | 0.01 | 0.72 | -0.40 | 0.54 | 0.52 |
| *imperv* |  | 0.63 | -0.36 | -0.63 | 0.65 | -0.20 | -0.39 |
| *agri* |  |  | -0.40 | -0.89 | 0.54 | -0.60 | -0.74 |
| *grass* |  |  |  | -0.02 | -0.34 | 0.28 | 0.24 |
| *shrub* |  |  |  |  | -0.50 | 0.49 | 0.71 |
| *per_strm* |  |  |  |  |  | -0.34 | -0.60 |
| *srr3* |  |  |  |  |  |  | 0.67 |

**Table S6.** Parameter estimates, standard errors, 95% confidence intervals and significance of fixed explanatory variables in one of the best-supported gravity models, used as predictors of gene flow. Model were chosen based on having the lowest AIC score and fit using restricted maximum likelihood. *w*: distance, *hli*: heat load index, *srr27*: large-scale topographic complexity, *imperv*: impervious surfaces, *srr3*: small-scale topographic complexity.

| Species | Parameter | β | SE | 95% CI | *t* |
| --- | --- | --- | --- | --- | --- |
| northern Idaho ground squirrel | Intercept | 4.45 | 2.99 |  | 1.38 |
|  | *w* | 0.14 | 0.02 | 0.1, 0.19 | 6.75 |
|  | *hli* | 0.79 | 0.36 | 0.08, 1.5 | 2.35 |
|  | *srr27* | -0.07 | 0.03 | -0.12, -0.01 | 2.44 |
| southern Idaho ground squirrel | Intercept | 8.1 | 2.48 |  | 3.27 |
|  | *w* | 0.19 | 0.04 | 0.11, 0.27 | 5.06 |
|  | *hli* | 0.66 | 0.24 | 0.22, 1.11 | 2.74 |
|  | *ffp* | 0.77 | 0.33 | 0.13, 1.4 | 2.34 |
|  | *imperv* | -0.05 | 0.02 | -0.09, -0.01 | 2.73 |
|  | *srr3* | 0.34 | 0.09 | 0.16, 0.52 | 3.74 |

**References**

Borgatti, S. P. and Everett, M. G. 1999. Models of core/periphery structures. - Soc. Networks 21: 375–395.

Cushman, S. A. and Landguth, E. L. 2010. Scale dependent inference in landscape genetics. - Landsc. Ecol. 25: 967–979.

Everett, M. G. and Borgatti, S. P. 2005. Extending centrality. - In: Carrington, P. J. et al. (eds), Models and methods in social network analysis. Cambridge University Press, pp. 57–76.

Freeman, L. et al. 1991. Centrality in valued graphs: A measure of betweenness based on network flow. - Soc. Networks 13: 141–154.

Garner, A. et al. 2005. Genetic diversity and population divergence in fragmented habitats: Conservation of Idaho ground squirrels. - Conserv. Genet. 6: 759–774.

Girvan, M. and Newman, M. E. J. 2002. Community structure in social and biological networks. - Proc. Natl. Acad. Sci. U. S. A. 99: 7821–6.

Goudet, J. 1995. FSTAT (Version 1.2): a computer program to calculate F-statistics. - J. Hered. 86: 485–486.

Hoisington-Lopez, J. L. et al. 2012. Species limits and integrated taxonomy of the Idaho ground squirrel ( Urocitellus brunneus ): genetic and ecological differentiation. - J. Mammal. 93: 589–604.

Nakagawa, S. and Schielzeth, H. 2013. A general and simple method for obtaining R2 from generalized linear mixed-effects models. - Methods Ecol. Evol. 4: 133–142.

Naujokaitis-Lewis, I. R. et al. 2013. Implications of incomplete networks on estimation of landscape genetic connectivity. - Conserv. Genet. in press.

Newman, M. E. J. 2006. Modularity and community structure in networks. - Proc. Natl. Acad. Sci. U. S. A. 103: 8577–82.

Raymond, M. and Rousset, F. 1995. GENEPOP (Version 1.2): population genetics software for exact tests and ecumenicism. - J. Hered. 86: 248–249.
